# Evidence-based development of school-based and family-involved prevention of overweight across Europe: The ENERGY-project's design and conceptual framework

**DOI:** 10.1186/1471-2458-10-276

**Published:** 2010-05-25

**Authors:** Johannes Brug, Saskia J te Velde, Mai JM Chinapaw, Elling Bere, Ilse de Bourdeaudhuij, Helen Moore, Lea Maes, Jorgen Jensen, Yannis Manios, Nanna Lien, Knut Inge Klepp, Tim Lobstein, Marloes Martens, Jo Salmon, Amika S Singh

**Affiliations:** 1 Department of Epidemiology & Biostatistics and the EMGO Institute for Health and Care Research, VU University Medical Center, Amsterdam, the Netherlands; 2 Department of Public & Occupational Health and the EMGO Institute for Health and Care Research, VU University Medical Center, Amsterdam, the Netherlands; 3 Faculty of Health and Sport, Agder University, Kristiansand, Norway; 4 Department of Movement and Sport Sciences, Ghent University, Ghent, Belgium; 5 Obesity related behaviours research group, Durham University, Durham, UK; 6 Department of Public Health, Ghent University, Ghent, Belgium; 7 Institute of Food and Resource Economics, University of Copenhagen, Copenhagen, Denmark; 8 Department of Nutrition and Dietetics, Harokopio University, Athens, Greece; 9 Department of Nutrition, University of Oslo, Oslo, Norway; 10 International Association for the Study of Obesity, London, UK; 11 Rescon, Amsterdam, the Netherlands; 12 Center for Physical Activity and Nutrition Research, Deakin University, Melbourne, Australia

## Abstract

**Background:**

There is an urgent need for more carefully developed public health measures in order to curb the obesity epidemic among youth. The overall aim of the "EuropeaN Energy balance Research to prevent excessive weight Gain among Youth" (ENERGY)-project is the development and formative evaluation of a theory-informed and evidence-based multi-component school-based and family-involved intervention program ready to be implemented and evaluated for effectiveness across Europe. This program aims at promoting the adoption or continuation of health behaviors that contribute to a healthy energy balance among school-aged children. Earlier studies have indicated that school and family environments are key determinants of energy-balance behaviors in schoolchildren. Schools are an important setting for health promotion in this age group, but school-based interventions mostly fail to target and involve the family environment.

**Methods:**

Led by a multidisciplinary team of researchers from eleven European countries and supported by a team of Australian experts, the ENERGY-project is informed by the Environmental Research Framework for Weight gain Prevention, and comprises a comprehensive epidemiological analysis including 1) systematic reviews of the literature, 2) secondary analyses of existing data, 3) focus group research, and 4) a cross European school-based survey.

**Results and discussion:**

The theoretical framework and the epidemiological analysis will subsequently inform stepwise intervention development targeting the most relevant energy balance-related behaviors and their personal, family-environmental and school-environmental determinants applying the Intervention Mapping protocol. The intervention scheme will undergo formative and pilot evaluation in five countries. The results of ENERGY will be disseminated among key stakeholders including researchers, policy makers and the general population.

**Conclusions:**

The ENERGY-project is an international, multidisciplinary effort to develop and test an evidence-based and theory-informed intervention program for obesity prevention among school-aged children.

## Background

In Europe as well as other affluent regions of the world, close to or more than half of the population is overweight or obese. Obesity is one of the main determinants of avoidable burden of disease [1]. In lack of affordable, non-invasive, long-term effective obesity treatment, and because the ill-health effects of obesity are not fully reversible, a stronger focus on obesity prevention has been advocated [2]. Because overweight and obesity in adulthood are predicted by childhood and adolescent overweight, obesity prevention should start early in life. One important target group is school-aged children, i.e. the age-group right before and at the start of adolescence. This is a risk age for unnecessary weight gain as well as an age in which children develop more behavioral autonomy and may make negative health behavior changes [3]. The need for obesity prevention in schoolchildren is further indicated by the prediction that in 2010 about 38% of school-aged children in the WHO European Region will be overweight, and more than one quarter will be obese. These figures are similar or even worse in other affluent regions, such as North America and Australasia [4]. This age group is certainly not the only age group of relevance for promoting a healthy energy balance. In fact energy balance should be promoted throughout the life course, and should start early in life and continue through adolescence, and adulthood. But such efforts should be tailored and targeted to the age group and their specific determinants. ENERGY focuses therefore on one important age group.

The school environment is regarded as a good setting for health promotion interventions among school-age children [5]. Schools offer an environment where almost all children can be reached repeatedly and continuously, and where health education can be combined with health promoting structural environmental changes. For obesity prevention, schools have additional relevance as a health promotion setting, because most children eat a significant amount of food at school and schools offer physical education as well as other physical activity opportunities [6].

Although genetic factors may influence the susceptibility of individuals to weight gain [7], there is consensus that changes in lifestyle behavior are driving the obesity epidemic [8] rather than changes in biologic or genetic factors. A long-term positive energy balance, i.e. energy input through food intake exceeds energy expenditure for body functions and physical activity, leads to storage of excess energy as fat, to weight gain, and eventually to obesity. Prevention of unnecessary weight gain should thus target modifiable behaviors that influence energy intake and expenditure. Obesity prevention among schoolchildren should therefore target dietary, physical activity and sedentary behaviors [2].

Findings of an increasing number of studies suggest that school-based interventions that target both sides of the energy balance are promising [9]. These studies also show, however, that although positive behavior changes are often induced, most of the interventions evaluated to date do not yet show success in reducing the prevalence of overweight, especially on the longer term [10]. Systematic reviews suggest that this is due to the fact that such intervention schemes were not always guided by a careful enough systematic theory and evidence-based development process; failed to combine both sides of the energy balance; did not combine educational with environmental change strategies; were not aiming or able to significantly involve the family and home environment; and did not conduct careful pretesting before larger scale implementation [10,11].

The ENERGY-project is aiming to learn from and update these reviews and apply a thorough epidemiological analysis prior to develop and test a school-based and family-involved intervention scheme. This intervention scheme is aimed at promoting dietary and physical activity behaviors that contribute to a healthy energy balance among school-aged children on the brink of adolescence.

The European Commission's Directorate General for Research funded the ENERGY-project, within its 7^th ^framework program (start date February 1^st ^2009). ENERGY stands for EuropeaN Energy balance Research to prevent excessive weight Gain among Youth. This paper describes the objectives, conceptual framework, methodologies and intended deliverables of the different parts of the ENERGY-project and its so-called work packages. We will first describe the specific objectives of ENERGY, followed by the planning model and conceptual framework, and the organization of the project. Finally, the work and research to be conducted and methods applied in the different planning and development steps are described.

## Objectives of the ENERGY-project

The overall aim of the ENERGY-project is the development of a theory- and evidence-based multi-component intervention scheme for prevention of unnecessary weight gain among school-aged children, ready to be implemented and tested for effectiveness across Europe. We aim to develop an intervention scheme that is both school-based and family-involved and promotes the adoption or continuation of specific health behaviors that contribute to a healthy energy balance. The primary target group of the ENERGY-project is children aged 10-12, i.e. youth in the transition between childhood and adolescence. Secondary intermediate target groups are their parents and school staff.

The specific objectives of the ENERGY-project are:

1. to perform a thorough, multidisciplinary analysis of the most important behaviors contributing to the energy balance of children, and their most important modifiable determinants, including personal as well as social-cultural, physical, and financial-economic environmental factors, with a specific focus on the family and school settings;

2. to identify successful intervention schemes and strategies as well as the factors mediating and moderating these successful schemes in different sub-populations based on age, gender and socio-economic status, including the exploration of financial intervention strategies that can be implemented in schools;

3. to design a multi-component school-based and family-involved intervention using obtained insights from the above mentioned analyses;

4. to test the multi-component intervention implemented for formative, process and intermediary outcome evaluation;

5. to prepare a large scale implementation and monitoring plan for dissemination of the intervention scheme.

To reach these objectives, we will work according to an established planning model. The tasks derived from that model were operationalized in eleven work packages.

## The planning model

**Figure 1:**
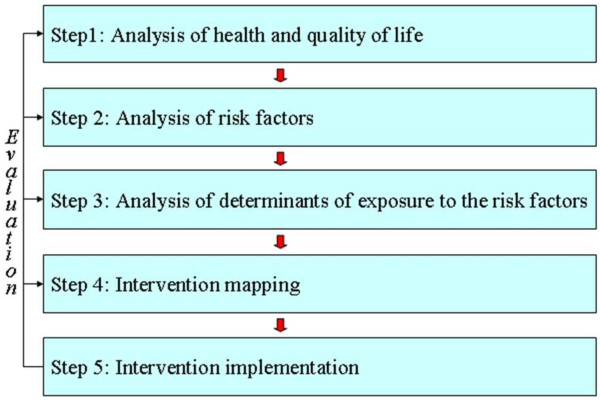
**A basic model of Planned Promotion of Population Health**.

In order to promote, induce and sustain meaningful changes in health behaviors, a planned stepwise approach has been advocated [12] (Figure 1).

The ENERGY-project follows the stepwise approach of the model of Planned Promotion of Population Health. ENERGY specifically focuses on steps 2, 3 and 4, i.e. the analyses of important and modifiable risk behaviors; of determinants of exposure to these risk behaviors; and on Intervention Mapping (IM). IM is the protocol through which the intervention is developed tailored to the identified risk behaviors and behavioral determinants, and prepared for implementation by means of formative evaluation and pilot testing in five countries [13].

### Step 2: Analysis of important risk behaviors

As outlined in the Background paragraph, unnecessary weight gain and eventually obesity is caused by a positive energy balance in which energy intake exceeds energy expenditure. Excessive energy intake through dietary behaviors relative to energy expenditure for maintaining bodily functions and engaging in physical activities are the risk factors at hand. Prevention of unnecessary weight gain should therefore aim at reducing energy intake by changes in dietary behaviors and increasing energy expenditure by changes in physical activity and sedentary behaviors. Different specific dietary, physical activity and sedentary behaviors may contribute to energy balance, and these behaviors have therefore been referred to as 'energy balance-related behaviors' (EBRB) [14]. It has been established that a combination of changes at both sides of the energy balance is the most promising strategy for successful obesity prevention [15].

Recent overviews have suggested a range of more specific EBRB that may contribute substantially to a higher risk for excessive weight gain. At the intake side of the energy balance, high intakes of energy-dense and/or micronutrient-poor foods, intakes of specific foods such as sugar-sweetened soft drinks, and large portion sizes may increase risk. Low energy expenditure because of a sedentary lifestyle has also been identified as associated with higher risk; TV viewing and computer time have been identified as specific sedentary risk behaviors. Although the evidence is not always consistent, especially where computer time is concerned [16], we choose to include both screen time behaviors as potential risk behaviors to be able to further contribute to the possible relevance and differences between both screen-time behaviors. Behaviors that may contribute to a lower risk of unnecessary weight gain are high dietary fiber intake and different physical activities, including sports participation, transport-related physical activity, and active play [2]. However, the evidence is not very strong, not always consistent [17] and the relevance of specific EBRB may differ between different age groups as well as according to gender, socioeconomic position and/or ethnicity.

There is thus need for a systematic review of the evidence for EBRB among school-aged children as well as further studies to examine the specific risk behaviors related to overweight and obesity.

### Step 3: Identification of determinants of exposure to the risk behaviors

In order to develop interventions to promote obesity prevention behaviors, insight into behavioral determinants is necessary. Each of the aforementioned EBRB may have different determinants; children's 'reasons' for drinking soft drinks may be different from their reasons for spending too much time watching television. Health behavior changes can be induced and sustained by targeting behavior-specific important and modifiable behavioral determinants [12].

Behavioral determinant studies have mostly been informed by psychological theories of human behavior. This has led to a strong focus on personal cognitive determinants such as attitudes, perceived control and motivation [18]. Health education has therefore been the primary tool to encourage the general public to adopt health-promoting lifestyles. Health education strongly focuses on conscious behavior change and on improving individuals' knowledge, attitudes, motivations and other cognitions that may increase the likelihood of adopting healthy behaviors. However, people's abilities and opportunities to make healthful behavior changes may be dependent on the environments in which they live [18]. It has been argued that obesity should be regarded as a normal response to an abnormal environment [19]. Effective promotion of EBRB therefore most probably requires changes in the environment to make the healthy choice the easy or even default choice.

Four 'types' of environments are often distinguished: physical, economic, political, and socio-cultural [19]. The physical environment refers to the availability and accessibility of opportunities for EBRB, such as availability of fruit and vegetables, soft drink vending machines, and sports facilities in the school and home environment. The economic environment refers to the costs related to healthy and unhealthy behaviors, such as the costs of soft drinks or fruit and vegetables, entrance fees to exercise facilities, et cetera. The political environment refers to the rules and regulations that may influence eating and physical activity and sedentary behavior. Bans on vending machines in schools or family rules on how many hours children can watch television are examples. The socio-cultural environment refers to the subjective and descriptive norms and other social influences such as parental support, demand or facilitation for adoption of health behavior, or social peer pressure to engage in unhealthy habits.

Recent studies show that it is not the personal cognitive factors *or *the environmental that is important, but rather the interplay between the two [14]. The environment we live in may influence our motivation, attitudes, perceived norms or control regarding EBRB. For example, an environment that offers plenty of opportunities for healthy food choices may improve motivation to eat a healthy diet, and may improve perceived abilities to eat healthily.

Kremers et al. [14] therefore argued that environmental factors may have a direct impact on EBRB, but these environmental influences are also likely to be mediated by individual-level factors. Kremers et al. further suggest that the causal pathway between environments or personal cognitions and EBRB may be moderated by such factors as personality, habit strengths and level of awareness of personal health behaviors. Based on these insights and preliminary evidence, Kremers et al proposed the Environmental Research framework for weight Gain prevention (EnRG-framework) which integrates potential personal cognitive determinants of EBRB with environmental factors and describes the hypothetical mediating and moderating pathways between cognitions and environments in influencing EBRB [14]. This EnRG-framework was informed by the Theory of Planned Behavior, i.e. a key social-psychological theory of health behavior, and the Analysis Grid for Environments Linked to Obesity (ANGELO), a model that details potential environmental determinants of EBRB [19].

**Figure 2:**
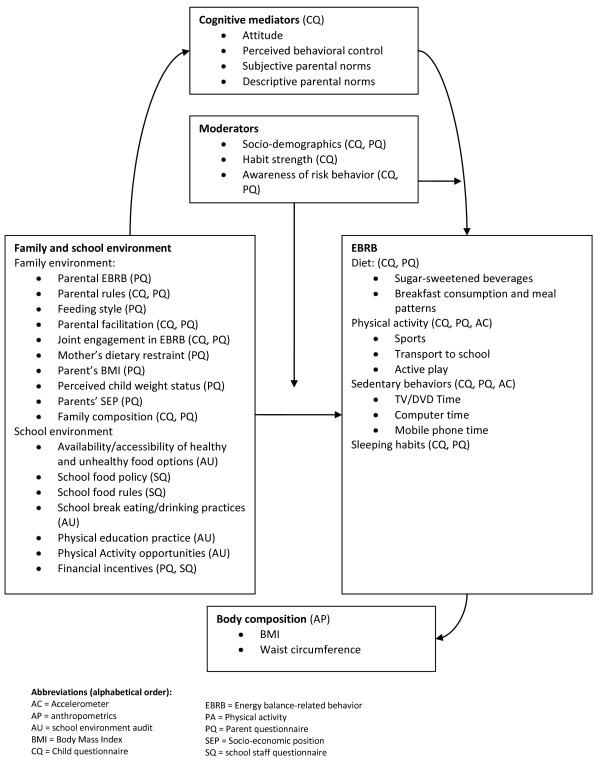
**The ENERGY-project specific EnRG (Environmental Research for weight Gain prevention) Framework**[14].

Recent reviews show that studies that explore mediating and moderating pathways between individual-level and environmental determinants are largely lacking, and such studies are especially needed to improve the evidence-base for EnRG. In the ENERGY-project we have adopted the EnRG framework with a specific focus on home and school environmental factors (Figure 2).

Within ENERGY this adapted EnRG framework will inform systematic reviews, secondary data analyses, original cross-European school-based survey research, and intervention development, formative and process evaluation research.

### Step 4: Intervention Mapping

The IM protocol introduced by Bartholomew and colleagues [13] suggests specific steps that guide problem-driven intervention development, supported by the application and integration of health behavior-change theories. IM proposes a systematic way to proceed from knowledge about behavioral determinants to specific change goals, and subsequently to intervention methods and strategies based on the production of intervention matrices. Such matrices finally develop into an 'intervention map' that makes the translation of objectives to change strategies to actual intervention activities explicit.

The ENERGY-project will use IM to translate the acquired insights in EBRB and their personal, family environmental and school environmental determinants into a school-based, family-involved intervention scheme that combines health education with environmental change strategies.

## Work plan and methods applied

**Figure 3:**
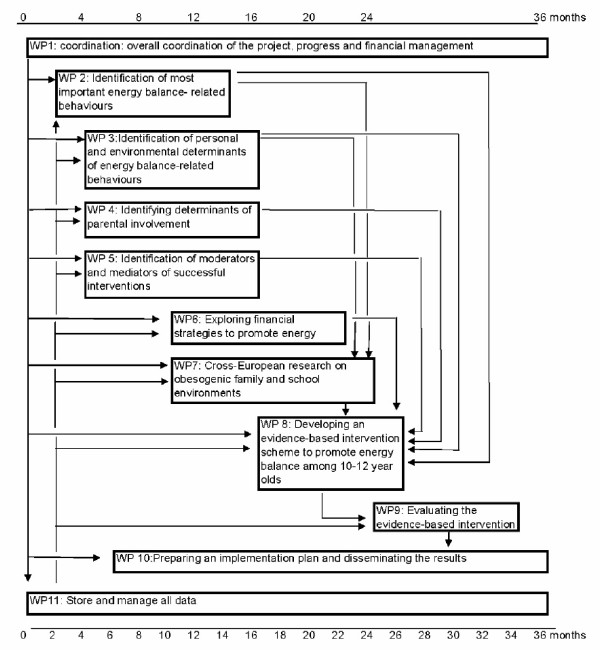
**Division of work and timeline of the ENERGY-project**.

In the ENERGY-project - in line with how European Commission-funded projects are organized - the work to go through the different planning steps is conducted in eleven work packages (WPs) (see Figure 3).

Within all WPs the same state-of-the-art methodology will be used: all WPs have a comparable structure according to which the timely finalization of all deliverables will be accomplished. We will conduct systematic literature reviews; secondary data analyses of existing data sets; focus group research; a cross-European school-based survey; and a school-based-preliminary trial to evaluate the newly developed intervention scheme. Finally, the results will be disseminated among key stakeholders, including researchers, policy makers and the general population.

**WP 1 **comprises the coordination task to promote and ensure integrated and timely progress of the ENERGY-project. This WP is responsible for the overall running and implementation of the project, will carry out the administrative tasks, and is responsible for the financial and organizational management of the project. This WP is also responsible for setting up the structure for communication through regular meetings and a website.

**WP 2 **identifies the most important EBRB among school children aged 10-12 years to contribute to step 2 of the planning model depicted in Figure 1 by reviewing the literature and conducting secondary data analyses of existing datasets.

**WP 3 **reviews the literature and conducts secondary data analyses to identify the most important personal, family environmental and school environmental correlates, predictors and determinants of the EBRB identified in WP 2 in order to contribute to step 3 of the planning model. Because most of the evidence on potential determinants of EBRB to date is based on cross-sectional data, WP2 and 3 will primarily focus on reviewing and re-analyzing longitudinal studies.

**WPs 4-6 **will identify successful intervention schemes, mediating pathways of intervention strategies, and important factors that moderate the effect of intervention strategies. These explorations are primarily based on secondary analyses of existing successful schemes promoting EBRB among schoolchildren and adolescents. WP 4 specifically addresses parental involvement in school-based schemes, by exploring what factors may promote parental involvement and what the impact of parental involvement on EBRB change in their children is. Earlier studies have indicated that parental factors and involvement is of key importance for school-aged children's EBRB and for effectiveness of primarily school-based interventions [20], but determinants of parental involvement in interventions have hardly been studied before. Therefore, this WP includes using qualitative methods, which are especially useful for exploring new research issues, as well as quantitative analyses of existing data. WP 5 explores what successful strategies or successful factors of ongoing intervention schemes consist of by means of a literature review and secondary data analyses. The review and secondary analyses will especially focus on mediation and moderation analyses to gain insight in the underlying processes of the intervention, in what determinants need to be addressed in the new intervention, and to explore what strategies work best according to, for example, gender, socioeconomic position or ethnicity. WP 6 explores economic strategies/incentives and whether these strategies can be effective and useful in school-based studies. Apart from a literature review, WP 6 makes an inventory of ongoing initiatives regarding economic incentives and further explores what are the possibilities to implement such strategies in the school setting by means of a survey among school staff and/or school board members. Economic strategies or incentives have hardly been used nor studied in school-based intervention schemes to date. In addition, WP 6 updates recent insights in the importance of marketing to children as a driving force behind the obesity epidemic especially related to family and school environments.

**WP 7 **comprises a cross-European survey on personal, family environmental and school environmental correlates of the different EBRB identified in WP 2. Europe has a relatively wide variety in social and physical environmental contexts, dietary practices, physical activity levels as well as rates of overweight and obesity, and the cross-European survey of WP 7 therefore offers the right framework for further exploration of potential determinants of EBRB and geographical differences in such determinants. The survey is conducted in seven countries (i.e. Belgium, Greece, Hungary, The Netherlands, Norway, Slovenia, Spain), located in different regions of Europe. After the ENERGY-project was approved, an eighth country - Switzerland - has joined the cross sectional survey WP of ENERGY with its own funding. This survey accumulates data on prevalence of overweight - based on objective measures of height, weight and waist-circumference -, on the most important EBRB identified in WP2, and on the most important correlates of these EBRB identified in WP 3, as outlined and detailed in Figure 2. This survey results in up-to-date data on EBRB and their personal and environmental correlates in different parts of Europe - including countries for which such data is as yet scarce or unavailable. It will provide intervention entry points for modification of specific EBRB and their determinants for WP 8.

**WP 8 **will carefully design the intervention using the IM protocol by using, integrating and translating information from WPs 2 -7 into specific change objectives, selection of effective strategies and development of intervention materials and protocols.

**WP 9 **is devoted to the pilot implementation and preliminary evaluation of the newly developed intervention in five different countries, again located in different regions of Europe: Norway, Germany, Belgium, Greece, and Hungary. The intervention will be implemented at five schools in each country and five other schools will serve as controls. The evaluation study will focus especially on process variables and intermediary outcomes.

**WP 10 **will use information from WP 9 to prepare the final intervention package, and an implementation, adoption, dissemination, and impact monitoring plan. WP 10 is also responsible for the further exploitation of the ENERGY-intervention scheme.

Finally, **WP 11 **files and administrates all data collected in the different WPs and oversees and guards quality-controlled data management and storage.

Different qualitative and quantitative methods are used in the WPs in ENERGY, including systematic reviews, focus group interviews, secondary analyses of existing data sets, as well as original survey and evaluation research.

### Reviews and secondary data analyses

The systematic reviews within WPs 2-6 are conducted according to a standard protocol. Basically, after a search strategy is developed and run, relevant papers are identified by subsequent selections of article titles, abstracts, and full papers, by two independent reviewers. Relevant data from the included studies is extracted and their methodological quality is scored, again independently by two reviewers. If possible and appropriate, the data of the retrieved studies are combined to conduct a formal meta-analysis.

Within WPs 2-5 analyses of existing datasets are conducted. Relevant datasets owned or managed by the partners in ENERGY will be used as well as additional potentially relevant datasets identified through the literature reviews. The secondary data analyses focus on mediation and moderation analyses of longitudinal data.

Although a number of school-based EBRB interventions have shown some effects, only very few evaluation studies have looked into how these effects came about, i.e. what the mediators of intervention effects were. Within the ENERGY-project the secondary data analyses will exactly do that: assess the mediators of intervention success or failure in order to gain insight in the underlying reasons why interventions may be effective or not. For these mediation analyses we will apply the procedure suggested by MacKinnon [21]. Furthermore, we will test moderators of intervention effects by including possible moderator X intervention interaction terms in the analyses to explore if interventions have differential effects according to, for example, gender, socio-economic position or ethnicity.

The study has been approved by the Medical Ethics Committee of Durham University.

### Original research

To explore what reasons parents have to be involved or not in their children's school-based health promotion interventions, a minimum of four focus group interviews will be conducted in each of four countries (Belgium, Norway, Spain, Hungary). Separate focus groups will be organized for one random group of parents and three separate groups with parents who (1) often participate in parent activities of school-based interventions; (2) parents with low socio-economic positions; and (3) parents with a low interest in EBRB. The focus groups will be conducted according to a standard protocol in all four countries. The interviews will be sound-recorded, and transcribed and submitted to content analyses.

The cross-European survey in WP7 will take place in schools in seven countries in the spring of 2010. We aim to collect data among approximately 7000 children to further study EBRB and their potential determinants according to region, ethnicity, gender and socio-economic status. The 1000 children per country will be recruited in a school-based manner. First three provinces in each country will be randomly selected. Within each province three municipalities with > 20,000 inhabitants will then be randomly selected. Lists of schools in these municipalities are obtained and schools will be randomly selected to be invited to participate. All children in the right school-classes for the study will be included in each school. WP 7 will administer survey questionnaires among schoolchildren, among their parents and school staff, and conduct audits of the school environment, and anthropometric measurements of the participating children. Children's height, weight and waist circumferences will be measured by trained staff, and accelerometers will be used to objectively assess physical activity in a sub-sample of about 600 children in three of the participating countries (Figure 2). All measurements will be performed according to written protocols and thus standardized between countries and schools. The research assistants responsible for the fieldwork receive a joint training.

The survey instruments used within WP7 will be developed based on the input of WPs 2-6, applying previously validated instruments where possible and appropriate. The survey questionnaires will be pilot-tested, evaluated for test-re-test reliability and internal consistency, and the pre-tested version will be translated to the languages of the other participating countries, and then back-translated for quality control.

The school-environment audit instrument will be adopted from the ENDORSE study [2].

Recruitment of schools, children and parents is conducted according to a standardized stepwise approach ensuring comparable samples in all countries, aiming for inclusion of lower and higher socio-economic position groups.

All data collected, i.e. completed surveys and audit instruments, reports on measured height, weight and waist circumference, and accelerometer records, will be sent to the coordinating center for data entry and data cleaning.

The evaluation study in the five countries will focus on comprehensibility, readability, relevance, credibility, and attractiveness of the intervention materials, next to preliminary evaluation of effects on EBRB and potential behavioral determinants. The effectiveness of the intervention is evaluated in a pre-test-post-test design, including an intervention and a control condition. This preliminary evaluation is conducted in a school-randomized controlled design. The intervention will be implemented in five schools, with two classes per school, in each of countries, and the results are compared to a five control schools in each country. Schools will be randomly allocated to intervention or control.

We aim for inclusion of approximately 2500 children in the preliminary evaluation study. Power calculations based on previous studies of school-based interventions indicate that this sample size is sufficient to detect relevant changes in EBRB, i.e. a 5% difference in change between intervention and control group, and their determinants [22].

Evaluation will be based on surveys among the participating children and their parents before and after the intervention has been implemented and on questionnaires completed by the school boards and the teachers in the intervention schools. The survey questionnaire and measurement protocols will be similar to those used in WP 7, but enriched with process evaluation questions.

## Discussion

The ENERGY-project applies the Intervention Mapping protocol informed by the Environmental Research Framework for Weight gain Prevention. The ENERGY-project aims to develop a school-based, family-involved intervention scheme to promote healthful EBRB in 10-12 year old school-aged children from countries located in different regions of Europe.

Potential strengths of the ENERGY-project are the multidisciplinary ENERGY consortium; the range of countries involved; the range of potential determinants of EBRB covered; as well as the stepwise development and testing of an intervention scheme informed by a mixed methods analysis of EBRB, their potential determinants and promising intervention strategies.

The ENERGY-consortium brings together a multidisciplinary team of experts on public health, epidemiology, human nutrition, physical activity, psychology, and health economics, totaling 14 partners, from 11 countries. These countries represent the North, West, South and Eastern parts of the European Union, including countries that lack data on EBRB and potential determinants among schoolchildren. The cross-European design of the ENERGY study will allow unique comparisons in EBRB and their correlates between countries and regions.

The fact that ENERGY focuses on a broad range of personal cognitive as well as family environmental and school environmental factors as potential determinants of EBRB is another potential strength of the study. Not many studies have done so in an international setting.

ENERGY will use different methods to carefully analyze which EBRB are most relevant; which behavioral determinants are supported by evidence; and which intervention strategies mediate intervention effects. The information from these analyses will inform the development of the ENERGY intervention scheme focusing on similar schemes in terms of intervention strategies and activities across countries, but where necessary tailored to the national situation. Such a stepwise approach is often not possible within the tight time-schedule of behavioral nutrition and physical activity intervention studies.

Another strength of ENERGY is the fact that we will have objectively measured weight, height and waist circumferences of the participating children, and objective assessments of physical activity using accelerometers in a sub-sample of children.

ENERGY also has several potential weaknesses or threats, and the time-schedule as well as the measurements are among these. The stepwise approach is only possible because the different steps are organized in different WPs, with many centers and researchers involved. Because the next WP is often partly dependent on the results of the previous one, working according to plan and the set timelines is of utmost importance. Although we obtain some objective measures, many of the measures taken are based on self-reports of children and their parents. Such self-reports may be liable to social desirability and recall bias. School-based surveys need to be completed in one school hour. The number of items that can be included in the questionnaire is therefore tightly restricted. Therefore, many of the potential determinants are assessed with few or even single-item measures, possibly reducing reliability.

A third weakness is the fact that the ENERGY survey is cross-sectional. This means that we will be able to explore correlates of EBRB, but not predictors or true determinants. Fortunately, the cross-sectional survey will be followed-up by an intervention study in which manipulation of the presumed determinants will be attempted, and a longitudinal design will be applied to test effects. A weakness of this intervention study is that we will be able to assess short-term results and intermediary effects only.

Nevertheless, we believe that the ENERGY-project with its cross-European approach is a unique endeavor to study EBRB, their potential determinants, and to develop and test an obesity prevention intervention scheme focusing on personal, family environmental and school environmental factors in different European countries.

## Competing interests

The authors declare that they have no competing interests.

## Authors' contributions

JB and StV designed the study at large, JB and ASS drafted the manuscript. All other co-authors designed different work packages of the ENERGY study and provided comments on the draft manuscript.

## Pre-publication history

The pre-publication history for this paper can be accessed here:

http://www.biomedcentral.com/1471-2458/10/276/prepub
